# Roles of Alternative Sigma Factors in Invasion and Growth Characteristics of *Listeria monocytogenes* 10403S Into Human Epithelial Colorectal Adenocarcinoma Caco-2 Cell

**DOI:** 10.3389/fmicb.2022.901484

**Published:** 2022-07-14

**Authors:** Junyaluck Rukit, Atsadang Boonmee, Teeratas Kijpornyongpan, Kan Tulsook, József Baranyi, Soraya Chaturongakul

**Affiliations:** ^1^Department of Microbiology, Faculty of Science, Mahidol University, Bangkok, Thailand; ^2^Department of Microbiology, Faculty of Science, Chulalongkorn University, Bangkok, Thailand; ^3^ Department of Botany and Plant Pathology, Purdue University, West Lafayette, IN, United States; ^4^National Center for Genetic Engineering and Biotechnology (BIOTEC), National Science and Technology Development Agency (NSTDA), Pathum Thani, Thailand; ^5^Institute of Nutrition, University of Debrecen, Debrecen, Hungary; ^6^Molecular Medical Biosciences Cluster, Institute of Molecular Biosciences, Mahidol University, Nakhon Pathom, Thailand

**Keywords:** *Listeria monocytogenes*, invasion, transcriptomics, RNA sequencing, Caco-2 cell

## Abstract

*Listeria monocytogenes* is a Gram-positive facultative intracellular bacterium with a broad host range. With its housekeeping sigma factor and four alternative ones (namely SigB, SigC, SigH, and SigL), *L. monocytogenes* can express genes in response to changing environments. However, the roles of these sigma factors in intracellular survival are still unclear. The objectives of this study were to characterize the role of each alternative σ factor on *L. monocytogenes* invasion and growth inside human epithelial colorectal adenocarcinoma Caco-2 cells. We used *L. monocytogenes* 10403S wild type and its 15 alternative sigma factor deletion mutants at a multiplicity of infection of 100 and 1 in invasion and intracellular growth assays in the Caco-2 cells, respectively. At 1.5, 2, 4, 6, 8, 10, and 12 h post-infection, Caco-2 cells were lysed, and intracellular *L. monocytogenes* were enumerated on brain-heart infusion agar. Colony-forming and growth rates were compared among strains. The results from phenotypic characterization confirmed that (i) SigB is the key factor for *L. monocytogenes* invasion and (ii) having only SigA (Δ*sigBCHL* strain) is sufficient to invade and multiply in the host cell at similar levels as the wild type. Our previous study suggested the negative role of SigL in bile stress response. In this study, we have shown that additional deletion of the *rpoN* (or *sigL*) gene to Δ*sigB*, Δ*sigC*, or Δ*sigH* could restore the impaired invasion efficiencies of the single mutant, suggesting the absence of SigL could enhance host invasion. Therefore, we further investigated the role of SigL during extracellular and intracellular life cycles. Using RNA sequencing, we identified 118 and 16 SigL-dependent genes during the extracellular and intracellular life cycles, respectively. The *sigL* gene itself was induced by fivefolds prior to the invasion, and 5.3 folds during Caco-2 infection, further suggesting the role of SigL in intracellular growth.

## Highlights

- Invasion and intracellular growth assays using *Listeria monocytogenes* wild type and 15 alternative sigma factor mutants allowed us to decipher the effects of bacterial alternative sigma factors on host intestinal epithelial cell infection.
- This information provides an understanding of gene regulation during the early infection of *L. monocytogenes*.
- We have confirmed the role of SigB during *in vitro* invasion and the role of SigA alone in maintaining abilities to invade and infect the host cells.
- RNA-seq analyses of wild-type and Δ*sigL* strains revealed genes *L. monocytogenes* expressed extracellularly and intracellularly, and SigL-dependent genes under these extracellular and intracellular conditions.
- Extracellular SigL regulon includes virulence-related factors, carbohydrate utilization, and transporters, while intracellular SigL regulon includes transporters of sugars and nucleic acid metabolism.
- Our finding provides novel insights into SigL-mediated gene regulation on metabolic pathways and bacterial responses inside the host cell.

## Introduction

*Listeria monocytogenes* is a foodborne intracellular pathogen that can survive in a wide host range (e.g., humans and ruminants). Being a facultative pathogen, *L. monocytogenes* is also able to grow in various non-host conditions, e.g., temperatures ranging from 1 to 45°C, salt concentrations up to 10% NaCl, and pH ranging from 4.4 to 9.6 (Membre et al., 2005; Goulet et al., 2013). Due to its, resistance to food preservation techniques, *L. monocytogenes* has a greater chance to be a food contaminant affecting humans and animals when ingested. *L. monocytogenes* poses a serious health risk because it can cause listeriosis in elderly persons, infants, pregnant women, and immunocompromised individuals. Novel strategies to control or eliminate *L. monocytogenes* contamination, such as the use of probiotics and natural antimicrobial peptides, are of wide interest as they could affect non-conventional targets in the stress persistent strains (Stincone et al., 2020; Margalho et al., 2021).

Human listeriosis manifestation ranges from mild conditions, such as gastroenteritis to severe septicemia, abortion in pregnant women, meningitis, and meningoencephalitis (Schlech, 2019; Charlier et al., 2020). When host animals ingest contaminated food, *L. monocytogenes* passes through the gastrointestinal tract and invade intestinal epithelial cells and phagocytic cells (Ray et al., 2009; Pizarro-Cerda et al., 2012). *L. monocytogenes* can enter the host cells by receptor-mediated endocytosis or phagocytosis (Ray et al., 2009; Pizarro-Cerda et al., 2012). To avoid reactive oxygen, nitrogen intermediates, and several lytic enzymes in phagolysosome, the bacterium can escape into the host cytosol using the pore-forming toxin listeriolysin O (LLO) and two phospholipase C enzymes (Kayal and Charbit, 2006; Ray et al., 2009; Hamon et al., 2012). Once within the host cytosol, *L. monocytogenes* mediates the actin polymerization by bacterial protein ActA and invades the neighboring cells.

To survive in the changing environments both outside and inside the host cell, *L. monocytogenes* alters its gene expression in response to stressors at the transcriptional level, specifically by reprogramming RNA polymerase-protomer recognitions. The associations between core RNA polymerase and dissociable sigma factors turn on the target gene sets. *L. monocytogenes* uses housekeeping and alternative sigma factor proteins to turn on the genes in response to various stress conditions outside and inside the host cell. There are four alternative sigma factors in *L. monocytogenes*, namely sigma B, C, H, and L that allow *L. monocytogenes* to survive under different environmental conditions (Chaturongakul and Boor, 2006; Chaturongakul et al., 2008, 2011). Alternative sigma factors control the previously identified virulence genes known to be under the master regulatory factor A (PrfA) (Kazmierczak et al., 2006; Scortti et al., 2007). The role of SigB has been well-demonstrated under various stress conditions and in virulence-associated characteristics outside the host cell, but not intracellularly (Chaturongakul and Boor, 2006). SigH has been shown to regulate *L. Monocytogenes* competence genes and intracellular growth (Medrano Romero and Morikawa, 2016). SigC is activated by heat stress (Zhang et al., 2005). The expression of SigL (or RpoN) is dependent on the growth phase and temperature (Liu et al., 2002; Arous et al., 2004; Chan et al., 2007). SigL in *L. monocytogenes* EGD-e strain is required for efficient growth under specific conditions, such as cold stress, acidic stress, and ethanol stress. Moreover, a *sigL* deletion mutant showed increased sensitivities to penicillin, tetracycline, and chloramphenicol (Mattila et al., 2012) and impaired growth under cold, salt, and acid stress conditions (Raimann et al., 2009). SigL-dependent genes have also been studied inside infected macrophage cell line P388D1 (Chatterjee et al., 2006).

Recently, high-throughput RNA sequencing techniques have identified *L. monocytogenes* σ^B^-dependent genes involved in stress response, pathogenesis, homeostasis, and resilience in enriched media outside the host cell condition (Liu et al., 2017). The σ^H^ has been shown to regulate *L. monocytogenes* competence genes in enriched media using the RNA sequencing technique (Zhang et al., 2005; Liu et al., 2016). Regulons of each alternative sigma factor and the overlapping regulons among alternative sigma factors and PrfA, CtsR, and HrcA have been shown in previous whole-genome microarray studies (Hu et al., 2007a,b; Chaturongakul et al., 2011). Based on these data, co-regulation by transcription factors has been demonstrated. Co-regulations of σ^B^ and the other sigma factors have been shown to control the largest group of genes involved in virulence and energy metabolism (Chaturongakul et al., 2011). Co-regulation between σ^H^ and σ^L^ has been shown to control (i) phosphotransferase system (PTS) that is important for extracellular growth at 37°C in *L. monocytogenes* strain 10403S (Stoll and Goebel, 2010; Wang et al., 2014), (ii) cell-division protein, pyrimidine-nucleoside phosphorylase, (iii) YktA protein similar to *B. subtilis*, and (iv) amino acid biosynthesis (Chaturongakul et al., 2011; Mujahid et al., 2013). Co-regulation between σ^B^ and σ^L^ has been shown to control propanediol utilization, branched-chain amino acid synthesis, and many PTSs during growth at 3 and 37°C (Mattila et al., 2020). Many studies have focused on the role of alternative sigma factors and the co-regulatory responses outside the host cell. However, such role and response of alternative sigma factors in *L. monocytogenes* during infection inside the human cells are still poorly understood. We hypothesized that alternative sigma factors and the interactions among alternative sigma factors play major roles in turning on the group of genes during *L. monocytogenes* infection and replication inside the host. To test this hypothesis, we used a human epithelial colorectal adenocarcinoma Caco-2 cell as a model and characterized the role of each sigma factor in *L. monocytogenes* invasion and growth inside the Caco-2 cell. Transcriptomic studies using RNA sequencing were used to identify the genes important for host invasion and bacterial propagation inside the host.

## Materials and Methods

### Bacterial Strains and Growth Conditions

All bacterial strains used in this study are listed in Supplementary Table 1. Prior to each experiment, wild-type (WT) *L. monocytogenes* strain 10403S and 15 deletion mutants (Wang et al., 2014) from −80°C freezer stocks were streaked on a Brain Heart Infusion (BHI) agar plate (Difco™, BD, United States) and incubated at 37°C overnight. The isolated colony from each strain was inoculated into 5-ml BHI broth (Difco™, BD, United States) for 37°C overnight (16–18 h), incubation with 200 rpm shaking. A 50 μl of overnight culture was inoculated into the new 5-ml BHI broth and incubated at 37°C with 200 rpm shaking until optical density at 600 nm (OD_600_) reached approximately 0.4, representing mid-log phase culture. A 500-μl aliquot of mid-log phase culture was inoculated into a new 50-ml BHI broth, and the synchronized culture was grown until the OD_600_ of 1 plus three additional hours at 37°C with 200 rpm shaking. This stage (OD_600_ + 3 h) was defined as a stationary phase with approximately 10^9^ colony forming units (CFU)/ml.

### Cell Line and Culture Conditions

Human colon carcinoma cell line (Caco-2) ATCC HTB-37 was used in this study. Caco-2 cell was cultured at 37°C and 5% CO_2_/95% air atmosphere in Dulbecco’s Modified Eagle’s medium (DMEM) (Gibco) was supplemented with 20% fetal bovine serum (FBS) (Gibco), 1% final concentration of 100X penicillin/streptomycin (Gibco), 1 mM of non-essential amino acids (Gibco), and 1 mM of l-glutamine (GlutaMAX, Gibco). The media were changed every 48–72 h by discarding old media and replacing it with new and complete DMEM.

### Invasion Assay

To perform the invasion assays (Supplementary Table 1), *L. monocytogenes* WT and 15 mutant strains were grown under the routine culturing method until the stationary phase as described above. Caco-2 cells were cultured and maintained in a T75-cm^2^ flask (Kim et al., 2005; Garner et al., 2006; Chaturongakul et al., 2011). Approximately 10^5^ Caco-2 cells were seeded into 24-well plates (Corning, United States) 5 days prior to infection. On the day of infection, two representative wells of Caco-2 cells were trypsinized and washed with phosphate-buffered saline (PBS) prior to enumeration. *L. monocytogenes* inoculum was prepared and used for infection at a multiplicity of infection (MOI) of 100 (100 bacterial cells: 1 host cell) for 30 min (Kim et al., 2005; Garner et al., 2006; Chaturongakul et al., 2011). The cells were washed three times with PBS and incubated with DMEM containing 150 μg/ml gentamicin (Corning Incorporated, Corning, NY, United States) to eliminate the extracellular bacterial cells. After 90 min, cells were lysed with cold, sterilized, distilled water. Intracellular viable bacterial cells were determined by plating serial dilutions on BHI agar. Invasion efficiency was calculated as the log_10_ number of bacteria recovered relative to the log_10_ number of bacteria used for inoculation. Experiments were performed in biological triplicates.

### Intracellular Survival Assay

Approximately 10^5^ Caco-2 cells were seeded onto a 24-well plate and incubated at 37°C with 5% CO_2_/95% air atmosphere for 96 h. Cells were infected with 16 strains of *L. monocytogenes* at MOI of 1 (1 bacterial cell:1 host cell) and incubated at 37°C with 5% CO_2_/95% air atmosphere for 30 min.

The cells were washed three times and incubated with DMEM containing 150 μg/ml gentamicin (Corning Incorporated, Corning, NY, United States). Cells were harvested at 2, 4, 6, 8, 10, and 12 h cells by lysing with cold, sterilized, and distilled water. Intracellular viable bacterial cells were determined by plating serial dilutions on BHI agar and reported as log CFU per well at each time point. Experiments were performed in biological triplicates. Doubling times (*T*_*d*_) were estimated from CFU/well values over the time points. The specific growth rates were calculated as μ = ln(2)/*T*_*d*_.

### *In vitro* Growth Assay

Overnight, cultures of *L. monocytogenes* strain 10403S and 15 deletion mutants were inoculated into 5-ml BHI broth and incubated at 37°C with 200 rpm shaking until OD_600_ of 0.4, representing mid-log phase culture. The mid-log cultures underwent five binary dilutions to prepare five inocula at approximately 10^5^, 5 × 10^4^, 2.5 × 10^4^, 1.25 × 10^4^, and 6.25 × 10^3^ CFU/ml in the final volume of 200 μl of BioScreen C Honeycomb microplate. The CFU/ml of inocula were plated on BHI agar for confirmation. Wells were loaded in duplicates and incubated at 37°C for 48 h. The OD_600_ values were recorded every 5 min. Experiments were performed in biological triplicates. The doubling time, *T*_*d*_, was calculated using the slope of the “detection times vs. number of dilutions” linear regression, where the OD detectable level was set to OD_*det*_ = 0.2 (Baranyi and Pin, 1999). The specific growth rates were calculated as μ = ln(2)/*T*_*d*_.

### Statistical Analysis for Phenotypic Characterization Assays

For all phenotypic characterizations (i.e., invasion assay, intracellular survival assay, and *in vitro* growth assay), data comparisons were analyzed using one-way ANOVA with Dunnett’s *post-hoc* test. Differences from *L. monocytogenes* WT with *p*-values of 0.05 were considered statistically significant.

### Extracellular Bacterial Total RNA Isolation and DNase Treatment

Stationary phase *L. monocytogenes* 10403S and isogenic deletion mutant Δ*sigL* were cultivated as described above. Approximately 10^8^ *L. monocytogenes* cells were inoculated into 26 ml of complete DMEM (Gibco) with 20% FBS (Gibco), 1 mM of non-essential amino acids (Gibco), and 1 mM of l-glutamine (GlutaMAX, Gibco). The bacterial cells in DMEM were incubated for 15 min at 37°C, and then 10% of the stop solution (Acid phenol pH 4.5: 100% ethanol) (Invitrogen™, United States) was added into the mixture. The mixture was kept at −80°C until used. Prior to total bacterial RNA isolation, the mixture was thawed on ice and then centrifuged at 14,000 × *g* for 20 min at 4°C to pellet the bacterial cells. The total RNA was extracted using the RiboPure™ Bacteria kit^®^ (Ambion, United States) according to the manufacturer’s instructions. The extracted total RNA was treated with the TURBO DNA-*free*™ DNase I treatment kit (Ambion, United States) according to the manufacturer’s protocol. The total RNA concentration and the quality were measured by Nanodrop (DeNovix, United States).

### Intracellular Bacterial Total RNA Isolation and DNase Treatment

Approximately 10^6^ Caco-2 cells were seeded into a T225 cm^2^ flask (Corning, United States) and incubated at 37°C with 5% CO_2_/95% air atmosphere for 5 days. WT and Δ*sigL* mutant *L. monocytogenes* strains were grown under the routine culturing method until the stationary phase stage. For *L. monocytogenes* infection, the day 5 Caco-2 cells were infected with stationary phase *L. monocytogenes* 10403S or Δ*sigL* mutant at MOI of 5 (5 bacterial cells:1 host cell) and incubated at 37°C with 5% CO_2_/95% air atmosphere for 30 min (Chatterjee et al., 2006; Joseph et al., 2006; Mraheil et al., 2011; Wehner et al., 2014; Schultze et al., 2015). After the washing steps, a freshly completed DMEM with 150 μg/ml gentamicin was added to eliminate the extracellular bacterial cell (Corning, United States). At 5.5 h after infection, infected monolayer cells were lysed with a cold lysing solution composed of 0.1% sodium dodecyl sulfate, 1% acidic phenol: chloroform, and 19% ethanol in a total volume of 20 ml. Total RNA was extracted using acid-phenol chloroform pH 4.5 (Invitrogen™, United States) as modified from the previous protocol (Pleitner et al., 2014). DNase treatment was performed using the TURBO DNA-*free*™ Dnase I treatment kit (Ambion, United States) according to the manufacturer’s protocol. Total RNA concentration and quality were measured by Nanodrop (DeNovix, United States).

### Host rRNA Removal Step for Intracellular Bacterial Total RNA Isolation

Host ribosomal RNA (rRNAs) and bacterial rRNAs were removed from total RNAs using the NEBNext rRNA (human/mouse/rat) depletion kit (New England Biolabs) according to the manufacturer’s instruction.

The RNA concentration and quality were measured by Nanodrop (DeNovix, United States).

### Real-Time Quantitative PCR

To check the quantity and the quality of RNA from the intracellular and extracellular RNA isolation steps, the RNA polymerase subunit beta *rpoB* gene was used as a target for real-time quantitative PCR (qRT-PCR). The copy number of *rpoB* gene was measured using the TagMan primers and probe as shown in Supplementary Table 2. TagMan primers and probes were designed by PrimerQuest (IDT DNA, Coralville, IA, Unites States) as previously reported (Boonmee et al., 2019). No template control samples have used nuclease-free water in place of the template. Reaction without the reverse transcriptase enzyme was used to identify gDNA contamination. Genomic DNA standard curves of *rpoB* gene were performed to determine the amplification efficiencies and to serve as positive controls in each assay. Dilutions of gDNA in 10^7^, 10^5^, and 10^3^ chromosomal copies were used as templates. Complementary DNA (cDNA) synthesis was performed at 48°C for 30 min followed by PCR setting of 1 cycle at 95°C for 10 min, 40 cycles at 95°C for 15 s, and 55°C for 1 min. The messenger RNA (mRNA) expression levels were log-transformed and compared to the standard gDNA.

To check the quantity and the quality of RNA from the intracellular RNA isolation step, a housekeeping *Homo sapiens* beta-actin (*ACTB*) gene was used as a target for two-step qRT-PCR. A total of 10 ng of RNA was converted into cDNA using a QuantiNova reverse transcription kit according to the manufacturer’s instructions. The 1:10 dilution of cDNA was used as the template of the qPCR step. The copy number of the *ACTB* gene was measured using the specific primer for beta-actin gene as shown in Supplementary Table 2, and the QuantiNova™ SYBR^®^ Green PCR kit according to the manufacturer’s instruction. No template control samples used the nuclease-free water in place of the template. Reaction without the reverse transcriptase enzyme was used to identify gDNA contamination. Genomic DNA standard curves of the beta-actin gene were performed to determine the amplification efficiencies and to serve as positive controls in each assay. Dilution of genomic human Caco-2 DNA in 100 ng, 10 ng, 1 ng, 100 pg, 10 pg, and 1 pg was used as templates. The mRNA expression levels were log-transformed and compared with the standard gDNA.

### RNA Sequencing and Data Analysis

RNA quality was assessed using Agilent 2100 Bioanalyzer system (Agilent Technology, Santa Clara, CA, United States) with the RNA 6000 Pico LabChip. The cDNA library was performed using a Trio RNA-Seq Library preparation kit (NuGEN). Paired-end sequencing was carried out on a NovaSeq 6000 Illumina platform as a service by Bangkok Genomics Innovation (China). Sequencing reads were mapped against *L. monocytogenes* 10403S (NCBI accession number: NC_017544.1). Reads were aligned and mapped with Bowtie2 and TopHat2 (Langmead and Salzberg, 2012; Trapnell et al., 2012). The cut-offs for percent mapped reads on either sense or antisense strand and percent of rRNA match rate were at least 75% and lower than 0.1%, respectively. The reads were counted by HTSeq-count (Anders et al., 2015). Differential gene expression was compared and analyzed using R program version 3.3.3 with the DESeq2 package (Love et al., 2014). Differential gene expression was considered when the False Discovery Rate (FDR) was less than 0.05 and the fold change was > 2 or < 0.5. Upregulated or downregulated genes were analyzed further with gene ontology (GO) enrichment analysis using R program version 3.3.3 with the GOseq package (Young et al., 2010). In brief, an unbiased selection test in differentially expressed vs. all genes in the genome of each GO term was performed and the significant value was inferred using the Wallenius approximation at *p*-value of < 0.05. GO based on the functional annotation of differential gene expression was grouped into three main categories: biological process, cellular component, and molecular function. The data sets generated or analyzed for this study were deposited and can be found with accession number: PRJNA761708.

## Results

### Roles of *Listeria monocytogenes* 10403S Sigma Factors in Human Intestinal Epithelial Cell Invasion

**FIGURE 1:**
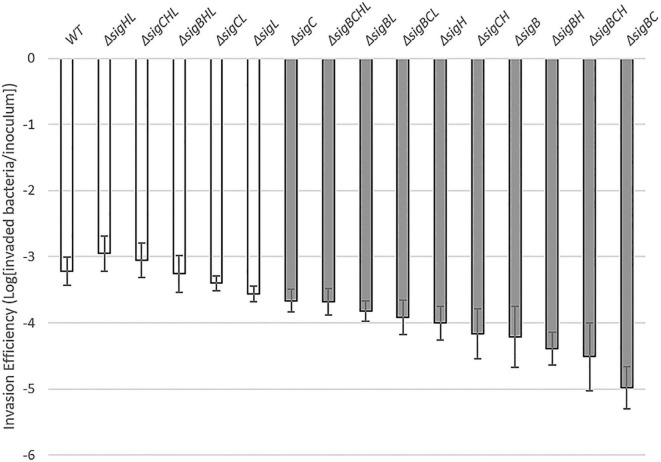
Caco-2 invasion by 16 *Listeria monocytogenes* strains. A invasion efficiency of each strain (*X*-axis) is shown in the log difference between invaded bacteria and inoculum of the corresponding strain (*Y*-axis). Experiments were performed in three biological replicates with two technical replicates each. The dark gray bar indicates a significant difference from WT based on one-way ANOVA with Dunnett’s *post-hoc* test at *p* ≤ 0.05. The white bar indicates a similar level of invasion efficiency as in WT.

First, we determined the Caco-2 invasion efficiencies of *L. monocytogenes* by calculating the log_10_ ratio of recovered bacteria to inoculated bacteria. For instance, the invasion efficiency for the *L. monocytogenes* wild-type strain 10403S was at −3.22 ± 0.21, indicating that at 1.5 h post-inoculation, approximately 10^4^ CFU (of 10^7^ CFU inoculum) could invade the host cells. From the invasion assay, we observed that all deletion mutants of *L. monocytogenes* could still invade the host Caco-2 cells because all deletion mutant strains still have the functioning housekeeping SigA (Figure 1). The invasion efficiency of the Δ*sigBCHL* strain (lacking all alternative sigma factors, but possessing the housekeeping SigA) was not significantly different from that of the wild-type (WT) strain. However, the ability to invade Caco-2 cells varied among mutant strains and combinations of gene deletions, with efficiencies ranging from −2.95 (Δ*sigHL*) to −4.97 (Δ*sigBC*). In other words, the abilities in host invasion among 16 tested strains vary by 2 logs depending on the remaining sigma factors in the bacteria. Single mutants lacking SigB, SigC, or SigH invade significantly less than WT. As expected from previous studies, the strains lacking SigB (e.g., Δ*sigB*, Δ*sigBC*, Δ*sigBH*, Δ*sigBL*, Δ*sigBCH*, Δ*sigBCL*, and Δ*sigBCHL*) invaded the Caco-2 cells with the lowest invasion efficiencies. The strains having a functioning SigB (e.g., Δ*sigL*, Δ*sigCL*, Δ*sigHL*, and Δ*sigCHL*) invaded the host cells at the same capacity as WT. SigB is confirmed as an important modulator in the regulation of receptor-mediated uptake and virulence of *L. monocytogenes* inside the human host cell. Surprisingly, the invasion efficiency of the Δ*sigBHL* strain was similar to WT and strains harboring SigB.

Among the single mutant, only Δ*sigL* did not show a significant impairment in invasion when compared to WT. In fact, most strains lacking SigL, except Δ*sigBCL* and Δ*sigBCHL*, invaded at the same level as WT. Additional deletion of *sigL* in Δ*sigB*, Δ*sigC*, or Δ*sigH* background, all of which showed a significant reduction in invasion efficiency, could improve invasion efficiencies. In other words, deletion of *sigL* could significantly repress the effect of SigB, SigC, and SigH in invasion. Given that (i) most strains with a deletion in *sigL* did not have any impairments in invasion, and (ii) *sigB* deletion alone or the combinations of *sigB* and *sigC* deletions (i.e., Δ*sigBL*, Δ*sigBCL*, and Δ*sigBCHL*) could reduce the invasion efficiency of null *sigL*, it is possible that a combination of SigL and the housekeeping SigA could significantly hamper the invasion into Caco-2 cell.

### Roles of *Listeria monocytogenes* 10403S Sigma Factors in Intracellular Growth Inside Caco-2 Cells

**FIGURE 2:**
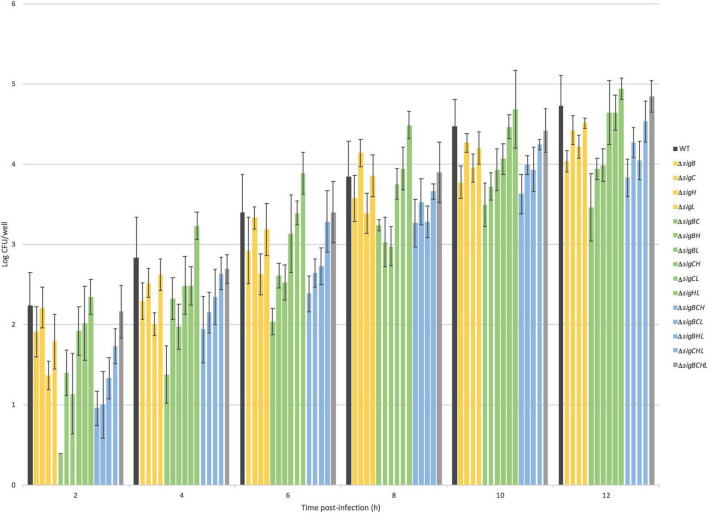
Intracellular growth of 16 *Listeria monocytogenes* strains. Numbers of intracellular *L. monocytogenes* are shown in log CFU/well (*Y*-axis). The *X*-axis shows time in hours post-infection. The dark gray bar represents WT 10403S *L. monocytogenes*, the yellow bars represent single deletion mutants, the green bars represent double deletion mutants, the blue bar represents triple deletion mutants, and the light gray bar represents quadruple mutant. The strains are ordered as seen in the legend on the right.

**FIGURE 3:**
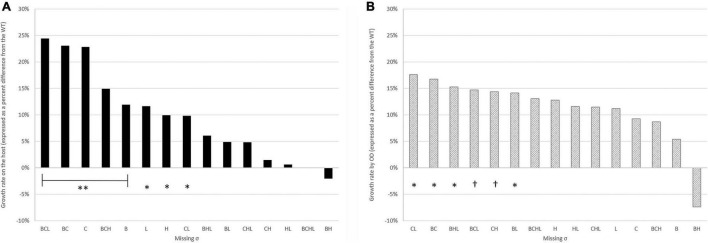
Growth rates on the Caco-2 host **(A)** and by OD measurement from BHI **(B)** expressed as percent differences from the WT. Median percentage values were used in the plot. **indicates significant difference from WT at *p* ≤ 0.01, *indicates significant difference from WT at 0.01 < *p* ≤ 0.05, and ^†^indicates *p* ≤ 0.07. All experiments were performed in biological triplicates.

To investigate the roles of sigma factors in the intracellular survival and replication of *L. monocytogenes*, we performed intracellular growth assays inside Caco-2 cells. CFU/well values of WT and its 15 mutants were enumerated at six time points post-infection as shown in Figure 2. All deletion mutant strains still have functioning sigma factors–either the remaining alternative sigma factors or the housekeeping SigA. As observed previously in our group that the quadruple mutant has the same phenotype as WT, the quadruple mutant intracellularly replicated at a similar level as WT over 12 h of observation. For strains lacking SigB, SigC, or SigH, deficiencies in invasion as shown earlier could hinder the ability to replicate intracellularly. Therefore, to better decipher the role of each sigma factor in intracellular replication, we considered the number of bacteria at 2 h as an inoculum of intracellular growth for each strain. Growth rates expressed as percent differences from the WT during exponential growth at 2–8 h post-infection (PI) were calculated and shown in Figure 3A. Data points from 10 and 12 h PI were omitted because, at these time points, cultures were entering the stationary phase. All single mutants grew 10–23% faster than WT. Among the double mutants, Δ*sigBC* (expressing SigH and SigL) grew the fastest at 23% in comparison to WT, while Δ*sigHL* (expressing SigB and SigC) and Δ*sigBH* (expressing SigC and SigL) grew the slowest at a similar level as WT. In addition, among the triple mutants, Δ*sigBCL* and Δ*sigBCH* (expressing SigH and SigL, respectively) were among the mutants growing the fastest in comparison to WT. However, absence of all alternative sigma factors in Δ*sigBCHL* did not result in any change in growth rate inside the host.

To assess whether these growth characteristics were only unique to the intracellular life cycle, all 16 strains were grown in brain heart infusion broth (BHI) at 37°C for 48 h. Growth was monitored from the recorded optical density at 600 nm. Growth rates of deletion mutants were compared to WT and reported in Figure 3B. In comparison to intracellular growth rates, growth in BHI showed fewer variabilities among all strains. Among the double mutants, Δ*sigCL* (expressing SigB and SigH) and Δ*sigBC* (expressing SigH and SigL) maintained their growth characteristics regardless of the environments (i.e., inside the host or in BHI). Δ*sigCL* and Δ*sigBC* grew significantly faster than the WT at 18 and 17%, respectively.

### Transcriptomic Analyses of Intracellular and Extracellular *Listeria monocytogenes* 10403S

Our phenotypic data have suggested that SigL and SigH could hamper the role of other σ factors during infection. Therefore, we first further investigated the role of SigL during intracellular growth using RNA sequencing on samples from intracellular wild-type and Δ*sigL* strains (i.e., triplicates of each or six total samples). The *T* = 6 h PI was chosen for RNA collection as this represented the mid-log phase of intracellular growth. RNA samples from extracellular wild type and Δ*sigL* in DMEM were also used to represent extracellular conditions (i.e., triplicates of each or six total samples). To assess the host mRNA contamination ratio, qRT-PCRs-targeting bacterial *rpoB* and host actin was performed. The ratio 10^6^ of host: 10^3^ of bacteria mRNA quantities was measured. The cDNA library was performed using a Trio Prokaryotic preparation kit with low total RNA input. Sequencing of 12 samples was carried out in one lane on a NovaSeq 6000 Illumina platform and the output of 150-base paired-end sequencing was 700 Gbps at 2,333 M reads or approximately 194 M reads per sample.

Sequencing reads were mapped against *L. monocytogenes* 10403S (NCBI accession number: NC_017544.1) for each of the strain/condition combinations. Differential gene expression was compared and analyzed using R program version 3.3.3 and DESeq2 package. Genes with differential expression were considered when the FDR was less than 0.05. Genes with fold change of >2 were called upregulated, while genes with fold change of <0.5 were called downregulated. A list of upregulated and downregulated genes is shown in Supplementary Tables 3–10. Four regulons were identified: (i) 745 differentially expressed genes (DEGs) when compared to extracellular and intracellular WT (Supplementary Tables 3, 4), (ii) 322 DEGs when compared to extracellular and intracellular Δ*sigL* (Supplementary Tables 5, 6), (iii) 16 DEGs when compared to intracellular WT and Δ*sigL* (Supplementary Tables 7, 8), and (iv) 118 DEGs when compared to extracellular WT and Δ*sigL* (Supplementary Tables 9, 10).

**FIGURE 4:**
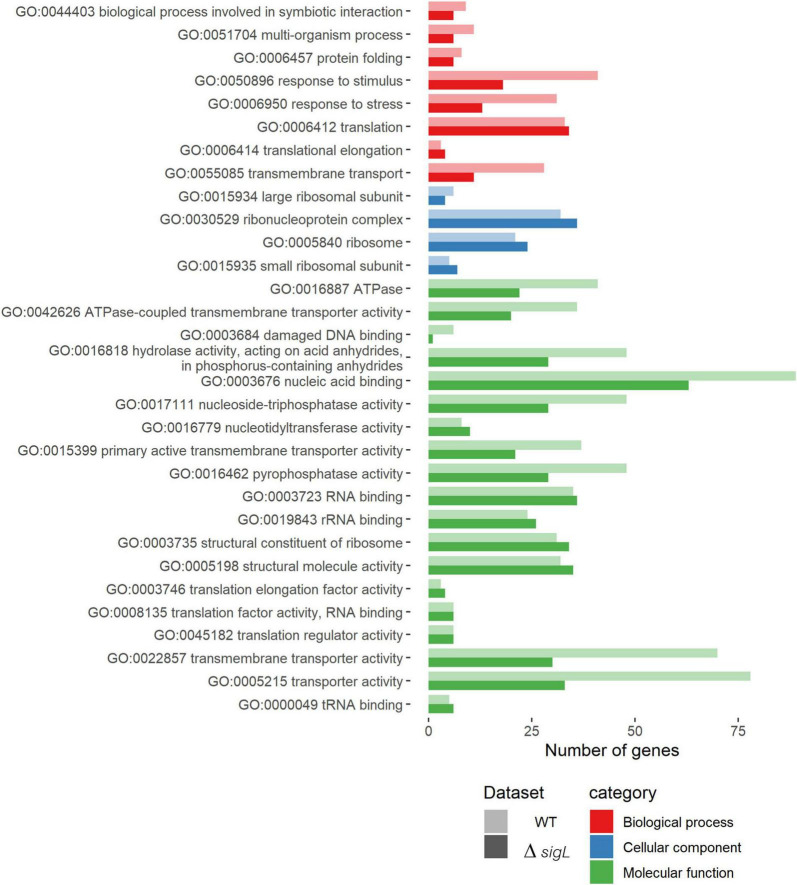
Enriched gene ontology (GO) terms in upregulated genes during intracellular growth of *Listeria monocytogenes* strain 10403S WT (light shade) and Δ*sigL* strain (dark shade). The *x*-axis represents the number of upregulated genes containing that GO term. Detailed information is shown in Supplementary Tables 4, 6.

To investigate the functional roles of SigL during the intracellular life cycle of *L. monocytogenes* in epithelial cells, GO enrichment analysis was performed. Among the enriched gene GO, upregulated genes during intracellular growth of WT and Δ*sigL* were grouped into three main categories: biological process, cellular component, and molecular function (Figure 4). Lacking SigL resulted in decreased expression of genes related to stress response, response to stimulus, transmembrane transport, damaged DNA binding, nucleic acid binding, hydrolase activity, nucleoside-triphosphatase activity, pyrophosphatase activity, primary active transmembrane transporter activity, transmembrane transporter activity, and transporter activity, especially for ATPase, the ATPs-coupled transmembrane transport activity.

**FIGURE 5:**
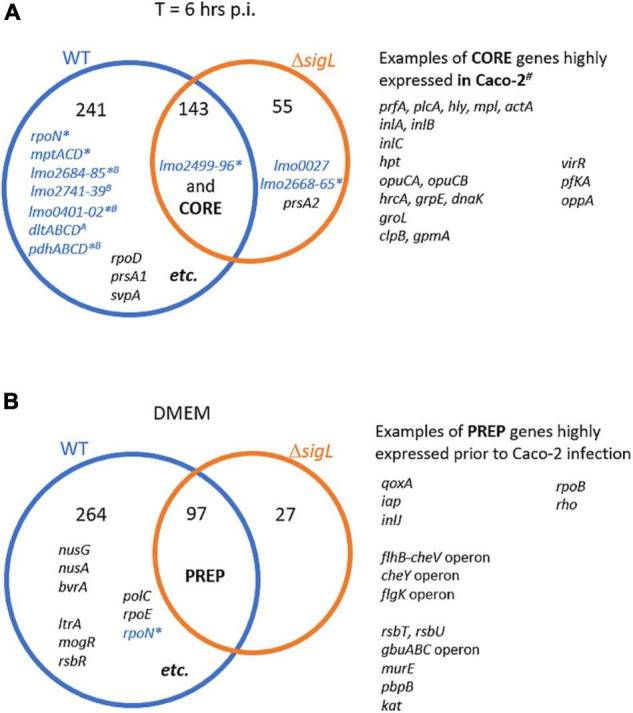
Differentially expressed genes (DEGs) during the process of Caco-2 infection. **(A)** Venn diagram showing numbers and examples of genes highly expressed inside Caco-2 during intracellular life cycles (*T* = 6 h PI). “Core” genes are highly expressed inside Caco-2 in both WT and Δ*sigL* strains. Detailed information is shown in Supplementary Tables 4, 6. Gene names in blue indicate they are SigL-dependent. * and *^B^*indicate gene/operon with putative SigL-dependent and putative SigB-dependent promoter, respectively (Chaturongakul et al., 2011). The ^#^indicates genes overlapping with those reported as intracellularly expressed inside Caco-2 cell line in the study by Joseph et al. (2006). **(B)** Venn diagram showing numbers and examples of genes highly expressed in DMEM as extracellular *Listeria monocytogenes* prior to *in vitro* infection. “Prep” genes are highly expressed in DMEM in both WT and Δ*sigL* strains. Detailed information is shown in Supplementary Tables 3, 5.

Among the upregulated DEGs during infection in WT and Δ*sigL* strains, we identified 143 overlapping genes, suggesting they are among the “core” genes expressed during intracellular replication (Supplementary Tables 4, 6 and Figure 5A). These core intracellularly expressed genes include the well-known virulence factor coding *prfA* and *virR* genes and their regulons. SigL-dependent genes differentially expressed in WT during infection include *sigL* itself, which was induced by 5.3-folds and operons-encoding sugar PTSs, such as *mptABC* and *pdhABCD* operons. Interestingly, using DMEM exposure as a representative condition for the extracellular environment allowed us to identify common preparation (or “prep”) genes, WT and Δ*sigL* strains, expressed prior to *in vitro* infection. Among the DE genes upregulated during DMEM exposure in WT and Δ*sigL* strains, we identified 97 overlapping genes (Supplementary Tables 3, 5 and Figure 5B). The *sigL* gene was also induced by fivefolds. Other prep genes include surface protein genes (i.e., *iap and inlJ*), stress response genes (i.e., *rsb* genes and *kat*), motility genes (i.e., *cheY* operon), and transcription process genes (i.e., *rpoB* and *rho*), suggesting key physiological state prior to infection.

## Discussion

The main goal of this study was to investigate the roles of alternative sigma factors and their potential interactions during *L. monocytogenes* infection. With the WT and 15 alternative sigma factor mutants, we initially performed invasion assays using the Caco-2 epithelial cells. Similar to the previous study (Chaturongakul et al., 2011), among the four single deletion mutants, Δ*sigB* mutant showed the lowest invasion efficiency that was significantly different when compared to the WT, confirming that SigB is a key regulator of *L. monocytogenes* invasion. Invasion assays have also shown that the other strains lacking SigB (i.e., Δ*sigBC*, Δ*sigBH*, Δ*sigBL*, Δ*sigBCH*, Δ*sigBCL*, and Δ*sigBCHL*) invaded Caco-2 cells at low efficiencies. One exception was observed in the Δ*sigBHL*, which had invasion efficiency at a similar level to WT, suggesting that presence of SigC and the housekeeping SigA were sufficient to maintain WT level entry. On the other hand, four of seven strains expressing SigB (i.e., Δ*sigHL*, Δ*sigCHL*, Δ*sigCL*, and Δ*sigL*) have demonstrated similar phenotypes as WT.

In this study, we also demonstrated the interaction among all four alternative sigma factors during *L. monocytogenes* invasion into human Caco-2 cell. Overall, the presence of SigL could reduce invasion efficiencies, as seen with Δ*sigBCH* strain expressing SigA and SigL among those with the lowest invasion efficiencies into the human Caco-2 cells. Interestingly, the loss of SigH and SigL (Δ*sigHL*) was negligible in terms of *L. monocytogenes* invasion efficiency in comparison to WT, while the loss of SigB and SigC (Δ*sigBC*) resulted in the lowest invasion efficiency. The qRT-PCR confirmation on *L. monocytogenes* internalin gene (*inlA*) expression supported this observation in that Δ*sigB*- and Δ*sigBC*-expressed *inlA* at approximately 4-5 logs less than WT and Δ*sigHL* (data not shown). This finding suggests preferred pairing among sigma factors during Caco-2 invasion, i.e., SigB/SigC vs. SigL/SigH combinations. Our recent study also revealed that the interaction of σ^H^ and σ^L^ decreased resistance to bile (Boonmee et al., 2021).

A previous study reported that, at 7.5 h post-inoculation, intracellular growth characteristics of *L. monocytogenes* 10403S and strains lacking one alternative sigma factor, i.e., Δ*sigB*, Δ*sigC*, Δ*sigH*, and Δ*sigL*, in LPS-activated J774 macrophages were similar (Chaturongakul et al., 2011). In this study, however, we demonstrated intracellular replications of *L. monocytogenes* 10403S and its 15 isogenic alternative σ factor mutants in Caco-2 cells. Throughout the assay, single mutants showed increased specific growth rates in comparison to WT with Δ*sigC*, having the highest specific growth rate. On the contrary, Δ*sigBH* was the one showing the least specific growth rate. The same phenotype was observed in Δ*sigBH* when grown in the rich medium BHI. In comparison to *in vitro* growth in BHI, we demonstrated that Δ*sigC*, Δ*sigBC*, Δ*sigBCH*, and Δ*sigBCL* strains had higher median-specific growth rates during intracellular growth. This finding, again, suggests the important roles of the remaining sigma factors, particularly SigH and SigL, in enhancing growth inside the host. On the other hand, if we further investigated the maximum number of each *L. monocytogenes* strain inside human Caco-2 cells, at entry into the stationary phase, the Δ*sigBC* had the lowest intracellular bacterial numbers, while the Δ*sigHL* had the highest intracellular bacterial numbers. The overall finding supports the preferred pairing between SigB/SigC vs. SigL/SigH combination.

Multiple studies have reported the highly expressed genes during infection both *in vitro* and *in vivo* (Chatterjee et al., 2006; Joseph et al., 2006; Camejo et al., 2009; Schultze et al., 2015). To gain more insight into the potential negative role of SigL during *L. monocytogenes* infection, transcriptome comparisons of the Δ*sigL* mutant and WT 10403S were conducted using *L. monocytogenes* cultures cultivated under both extracellular conditions in DMEM and intracellular conditions inside Caco-2 cell at 37°C. Four regulons were identified: (i) 745 DEGs when comparing extracellular and intracellular WT, (ii) 322 DEGs when comparing extracellular and intracellular Δ*sigL*, (iii) 16 DEGs when comparing intracellular WT and Δ*sigL*, and (iv) 118 DEGs when compared extracellular WT and Δ*sigL*.

The extracellular and intracellular transcriptomes of *L. monocytogenes* WT and Δ*sigL* were identified when we compared gene expression profiles of each strain growing in the DMEM condition and growing inside the Caco-2 cells, respectively. Differential gene expression was considered when the FDR was less than 0.05 and the fold change was > 2 or < 0.5. A total of 384 genes were identified as upregulated intracellularly in WT, and 198 genes were upregulated intracellularly in Δ*sigL*. As expected, virulence genes of *L. monocytogenes* under the positive regulator factor (PrfA), namely *prfA* itself, *plcA*, *actA*, *hly*, *mpl*, *inlAB*, and *inlC* genes were upregulated intracellularly in both strains. In addition, stress response genes such as *hrcA* operon encoding heat shock response proteins and HrcA-SigB co-regulon (e.g., *dnaK*, *groL*) (Hu et al., 2007a) were up-regulated intracellularly in both strains. The *hpt* (*LMRG_02261* or *lmo0838*), encoding a hexose phosphate transporter required for intracellular growth of *L. monocytogenes*, and the *opuC* operon encoding a carnitine transporter, involved in the osmoregulation of *L. monocytogenes*, was upregulated intracellularly. We confirmed that such genes can be called “core” intracellular life cycle genes in *L. monocytogenes*. Among the 143 core genes that we identified, 55 overlapped with those reported by the microarray technique of Joseph et al. (2006).

Among the important core genes for intracellular life cycle and genes expressed by WT during intracellular life in Caco-2, we identified those that are regulated by SigL, either based on RNA-seq results in this study or when compared to those having putative SigL promoter (Chaturongakul et al., 2011). We confirmed the *mptACD* (*lmo0096-0098*) operon encoding a mannose/fructose/sorbose PTS, previously characterized as SigL-dependent using microarray, proteomic studies, and transcriptomic profiles under BHI and bile (Arous et al., 2004; Mattila et al., 2012; Mujahid et al., 2013), is also induced inside the Caco-2 cells. In addition, we identified many other SigL-dependent operons encoding transport systems, namely the *lmo2499-96* (phosphate) operon, the *lmo2684-85* (cellobiose/lactose/fructose) operon, the *lmo2741-39* (multidrug efflux) operon, and the *lmo0401-02* (fructose) operon. Similar findings have been reported in that *mptACD* and *lmo2499-96* operons were upregulated in P388D1 murine macrophage (Schultze et al., 2015). Additionally, SigL also positively regulated the pyruvate dehydrogenase *pdhABCD* operon and the cell wall synthesis *dltABCD* operon during Caco-2 infection. Interestingly, these identified operons have been shown to be co-regulated by SigB, specifically *lmo2684-85*, *lmo2741-39*, *lmo0401-02*, and *pdhABCD* operons, suggesting the interactions between SigL and SigB during infection (Hain et al., 2008; Chaturongakul et al., 2011). Moreover, both *rpoN* and *rpoD* genes encoding SigL and SigA were upregulated in WT during infection and the *dltABCD* operon is co-regulated by SigL and SigA. Additionally, expression of the *dltABCD* operon was previously shown to be dependent on VirR, a two-component virulence response regulator (Mandin et al., 2005), whose corresponding gene was also identified in our study as one of the core intracellular genes. In summary, our RNA-seq data further demonstrated the potential interactions in gene expression among many factors such as SigL, SigA, SigB, and VirA in *L. monocytogenes* during infection.

Among intracellularly upregulated genes in Δ*sigL* strain, glucose utilization genes (e.g., *lmo0027* encoding a PTS beta-glucoside transporter subunit EIIBCA, *lmo2668-65* operon encoding galactitol PTS) were identified. Congruently, these genes were previously identified as negatively regulated by SigL (Chaturongakul et al., 2011; Mujahid et al., 2013), therefore, the absence of SigL could derepress glucose utilization inside Caco-2. Although *L. monocytogenes* grown in J774 murine macrophage cells and Caco-2 cells did not prefer glucose utilization as a major carbon substrate (Wang et al., 2014), SigL could play a role in balancing the expression of these genes. In addition to glucose utilization genes, expression of *prsA2* gene encoding protein chaperone for virulence factor PrsA2 (Alonzo et al., 2009) was also upregulated in Δ*sigL*. On the contrary, its homolog *prsA1* was upregulated in WT. Although we cannot conclude that upregulation of *prsA2* mRNA detected by RNA-seq from Δ*sigL* strain would function as a chaperone protein or as a regulatory RNA to *hly* mRNA during infection (Ignatov et al., 2020), this finding suggests the interplay between PrsA2 and PrsA1 expression levels in the presence or absence of SigL. Further investigation is needed to decipher the role of the effect of SigL on PrsA chaperone.

For an extracellular environment, DMEM was used to represent the condition. Alternatively, we can consider this condition as an environment that *L. monocytogenes* might encounter prior to *in vitro* adhesion and invasion. A total of 361 genes were identified as upregulated extracellularly in WT and 124 genes were upregulated extracellularly in Δ*sigL*. Of these upregulated genes in DMEM in WT and Δ*sigL*, 97 genes overlapped. We highlight that these genes can be taken a closer look and considered as preparation (or “prep”) genes for infection. These prep genes encode (i) surface proteins (e.g., QoxA, Iap, InlJ), (ii) motility proteins (e.g., flagellar and chemotaxis proteins from *flhB*, *flgK*, and *cheY* operons), (iii) stress-response proteins (e.g., regulators of SigB, catalase), and (iv) transcription proteins (e.g., RNA polymerase subunits, termination proteins). Among the upregulated key genes in WT in DMEM, the same four categories were identified suggesting the importance of coordinating the expression of genes in these categories. Interestingly, gene-encoding regulators of SigB or Rsb proteins, specifically *rsbSTU*, were upregulated in WT, and only *rsbTU* were regulated in Δ*sigL*. This finding not only confirms the involvement of SigB as a preparation or a required factor prior to infection but also suggests an involvement of *L. monocytogenes* stressosome and RsbS:RsbT/RsbT:RsbU partner switching in σ^B^ activation pathway (Guerreiro et al., 2020).

## Conclusion

In summary, this study has demonstrated that interactions among sigma factors are very important for *L. monocytogenes* invasion and growth inside the host cells. Although having only SigA (Δ*sigBCHL* strain) is sufficient to invade and multiply in the host cell at similar levels as the wild type, SigB is still the key factor for *L. monocytogenes* invasion. Our data further suggest working preferences among sigma factors. Having five sigma factors in *L. monocytogenes* (i.e., SigA, SigB, SigC, SigH, and SigL) meant, in certain conditions, preference for sigma factor pair/group working well together in response to the environment. Under invasion and intracellular growth conditions in the Caco-2 cell, we propose that SigB and SigC play positive roles in *L. monocytogenes*, while SigL and SigH could play the negative roles. SigB and SigL seem to exhibit a see-saw model in balancing gene set expression. RNA-seq data (i) confirmed genes important for the intracellular life cycle of *L. monocytogenes*, (ii) identified gene sets expressed during infection that are SigL-dependent and that suggested interactions between SigB and SigL, and (iii) suggested preparation or marker gene sets expressed prior to infection. Based on our results, further investigations could be carried out to decipher the mechanism of SigB-SigL interaction on intracellular gene sets (e.g., *pdhABCD* operon) and to better design anti-listerial compounds targeting SigB and SigL.

## Data Availability Statement

The datasets presented in this study can be found in online repositories. The names of the repository/repositories and accession number(s) can be found below: NCBI SRA-SRR15814244–SRR15814255.

## Author Contributions

JR and SC performed the experiments, analyzed the data, and prepared the manuscript. AB, TK, and KT supported the RNA-sequencing and data analyses. JB supported the growth characteristics analyses. SC conceived the study. All authors approved the final version.

## Conflict of Interest

The authors declare that the research was conducted in the absence of any commercial or financial relationships that could be construed as a potential conflict of interest.

## Publisher’s Note

All claims expressed in this article are solely those of the authors and do not necessarily represent those of their affiliated organizations, or those of the publisher, the editors and the reviewers. Any product that may be evaluated in this article, or claim that may be made by its manufacturer, is not guaranteed or endorsed by the publisher.
